# Systematic Review of Accuracy Differences in NIPT Methods for Common Aneuploidy Screening

**DOI:** 10.3390/jcm14082813

**Published:** 2025-04-18

**Authors:** Tamas Marton, Zsófia R. Erdélyi, Minori Takai, Balázs Mészáros, Dorina Supák, Nándor Ács, Zoltán Kukor, Zoltan Herold, Beata Hargitai, Sándor Valent

**Affiliations:** 1 Department of Obstetrics and Gynecology, Semmelweis University, 1082 Budapest, Hungary; marton.tamas@semmelweis.hu (T.M.); erdelyi.zsofia@stud.semmelweis.hu (Z.R.E.); minori990628@gmail.com (M.T.); valent.sandor@semmelweis.hu (S.V.); 2 Institute of Biochemistry and Molecular Biology, Department of Molecular Biology, Semmelweis University, 1094 Budapest, Hungary; 3 Division of Oncology, Department of Internal Medicine and Oncology, Semmelweis University, 1083 Budapest, Hungary

**Keywords:** NIPT, non-invasive prenatal testing, cfDNA, common trisomy screening, socioeconomical cost, prenatal diagnosis

## Abstract

**Background/Objectives:** Non-invasive prenatal testing (NIPT) has become a widely used method for screening common fetal aneuploidies due to its high sensitivity and specificity compared to traditional screening methods. With various NIPT technologies available, such as whole-genome sequencing (WGS), single nucleotide polymorphisms (SNPs), microarray, and rolling circle amplification (RCA), understanding the accuracy and reliability of each method is critical for clinical decision-making. However, comprehensive evaluations comparing the performance of these NIPT methods, especially in terms of predictive values for trisomy detection, remain limited. A systematic review of the difference in accuracy of the different NIPT methods used for common aneuploidy screening. **Methods:** A systematic review of former clinical studies using different NIPT methods, such as whole-genome sequencing (WGS), a targeted method of single nucleotide polymorphisms (SNPs), microarray and rolling circle amplification (RCA). We collected data from the PubMed, Embase, Web of Science, Scopus, clinicaltrials.gov, and Cochrane library from the last 20 years, between 2003 January and 2023 October, without any language, search filter or publication type restrictions. **Results:** Two authors selected twenty articles including twenty-one studies to perform the systematic review. In these studies, altogether 92,164 pregnant women were tested by genomics-based non-invasive prenatal testing (NIPT). We extracted data on true positive, false positive, false negative, and true negative values from each study, and calculated sensitivity, specificity, positive predictive value (PPV) and negative predictive value (NPV) from them. We collected data regarding trisomy 21 (T21), trisomy 18 (T18) and trisomy 13 (T13) detection from all studies. **Conclusions:** As a conclusion, for the detection of common fetal trisomies, different methods of NIPT perform similarly in terms of clinical sensitivity, specificity and NPV. However, the tests utilizing SNP and RCA had lower PPV values than other NIPT methods. Our research indicates all NIPT methods showed greater sensitivity for the detection of T21, above 97%, than traditional screening tests. For T18 detection, the targeted method with the microarray had a lower sensitivity compared to other tests. The SNP and the microarray-based test had high PPV, whilst the other tests, utilizing WGS, and the test with RCA had quite low PPV. Regarding T13 detection, all of the tests performed similarly in terms of clinical sensitivity, specificity, PPV, and NPV (with one exception—one of the tests using WGS had lower PPV). According to these results, there was no significant difference between the methods of NIPT, such as WGS, SNPs, microarray, and RCA, used to detect common trisomies, but the variation in PPV underlines the importance of invasive tests to derive positive NIPT results. We suggest that NIPT combined with US screening for structural abnormalities could further improve the utility of the non-invasive tests in pregnancy. This is the first independent systematic review into the efficacy of the different NIPT methods.

## 1. Introduction

Numerical chromosomal anomalies trisomy 21, trisomy 18, and trisomy 13 are the most frequent causes of fetal abnormalities. Early detection of these is crucial for managing affected pregnancies.

Non-invasive prenatal testing (NIPT) in general utilizes fetal cell-free DNA (cfDNA) in maternal blood, which is derived from the placenta. The cfDNA-based screening methods demonstrate higher sensitivity (around 99%) in the detection of common aneuploidies, with significantly lower false positive rates and a higher positive predictive value compared to the conventional biochemical marker-based screenings. They are non-invasive, requiring only a maternal blood sample, and can be performed as early as 10 weeks’ gestation. cfDNA screening is regarded currently as the most effective method for screening chromosomal abnormalities.

The commercially available NIPT tests use different methods for detecting cfDNA in the maternal blood, including whole-genome sequencing (WGS), or targeted methods such as single nucleotide polymorphism (SNP) analysis, microarray analysis, or rolling circle amplification (RCA). The methods have their advantages and limitations, influencing their application in clinical practice. Understanding these nuances is essential when choosing the appropriate NIPT test.

WGS-based NIPT provides the most informative results via analysis of the complete genome. Therefore, WGS-based NIPT has lower failure rates compared to targeted sequencing or array-based platforms, due to its broader genome coverage.

Methods behind WGS based NIPT: In NGS, multiple DNA fragments are sequenced in parallel. Other names for this process are massively parallel sequencing, short-read sequencing, high-throughput sequencing, deep sequencing, and second-generation sequencing. In comparison, Sanger sequencing replicates the sequence of large DNA fragments, while NGS results in exponentially larger amounts of DNA at significantly reduced costs and also provides faster sequencing. Unfortunately, WGS-based NIPT technologies remain more expensive than targeted approaches. The rapidly dropping costs of sequencing, combined with the ability to produce large volume of data using today’s sequencers, make whole-genome sequencing a powerful tool for genomics research. The use of PCR-free sample preparation in WGS-based NIPT enhances laboratory workflow, decreases assay complexity, and significantly reduces turn-around time (TAT). And finally, because of its advantages, the NGS technology for NIPT is highly adaptable, making it well-suited to meeting the requirements of a maturing laboratory [1,2].

Targeted technologies for NIPT include SNP analysis, microarray, and RCA. These approaches focus on specific regions of the chromosomes. The disadvantage of these technics is that they require additional steps and multiple rounds of amplification compared to whole-genome sequencing methods, leading to a more complex workflow.

SNPs are genetic variations that can be used to detect differences between parental and offspring DNA, as well as inferring copy number variations, which is used for NIPT. cfDNA is amplified using the PCR method with specific SNP targets, sequenced, and analyzed to identify abnormalities in allele frequencies [3].

With the microarray concept, DNA regions are affixed to a solid surface matrix. Amplified cfDNA fragments are then fluorescently labeled and bind to complementary sequences on the NIPT microarray plate. Light intensity and binding positions indicate DNA quantity and target presence, with anomalies suggesting aneuploidy [4,5].

RCA targets specific cfDNA fragments, which bind to a circular template and undergo replication. The replication products are fluorescently labeled, and deviations in fluorescence intensity indicate aneuploidy [6]. The advantages of RCA are that it is simple, it eliminates PCR and NGS, and it has reduced installation, hands-on time, bioinformatics, and running costs. Finally, this technology offers cost savings for medical systems due to its lower no-call rate compared to sequencing [7].

NIPT may be offered after initial screening but before diagnostic testing to more accurately identify pregnant women at high risk for fetal aneuploidy, [8]. NGS-based methods have been proposed before, as alternatives to traditional first-tier screening tests, including ultrasound and biochemical techniques. Furthermore, some even suggest NGS-based assays could replace diagnostic procedures like karyotyping fetal cells obtained through chorionic villus sampling (CVS) or amniocentesis [9]. Currently, in many countries, the most cost-effective option, particularly for publicly funded programs, is to use NIPT as a secondary screening test. While previously deemed economically inefficient as a primary test [10], its cost-effectiveness has recently improved. It is recognized that NIPT has a higher failure rate compared to conventional prenatal screening methods [8] and cannot detect all chromosomal abnormalities or adverse obstetric outcomes. Therefore, with the common, pragmatic approach, it complements maternal serum screening, ultrasound, and invasive diagnostic tests. The rationale is that NIPT can detect common aneuploidies, which only account for 44–64% of all chromosomal abnormalities identified during prenatal diagnosis [11,12].

### 1.1. Implications for Research

The new genomics-based NIPT approach is marketed as having higher sensitivity and lower false positive rates than traditional screening tests. When used as a second-tier screening test, NIPT enhances the detection specificity for fetal aneuploidies (T21, T18, and T13) and minimizes invasive procedures. Limited data from comparative studies also suggest it has significantly better specificity than traditional methods using maternal serum biochemical markers, ultrasound, or both [13,14,15]. Most previous studies overlooked the differences between commercially available NIPTs, and did not distinguish between the genetic analysis methods used. Our systematic review highlights the need for well-designed, large-scale, independent studies comparing different NIPT approaches to validate their accuracy. Lastly, systematic reviews are essential to broaden the understanding of NIPTs’ clinical utility across various settings. We focused our research on commercially available labels for simplicity, in order to help derive the implications for clinical application.

### 1.2. Objective

In this study, we performed a systematic review comparing the sensitivity, specificity, and efficacy of various available cfDNA screening methods, utilizing WGS and targeted approaches.

### 1.3. Review Question

We sought to evaluate the diagnostic accuracy of commercially available non-invasive prenatal tests (NIPT) for detecting trisomies (T21, T18, and T13) by synthesizing sensitivity, specificity, positive predictive value (PPV), and negative predictive value (NPV) metrics from published studies.

## 2. Materials and Methods

### 2.1. Test Selection

The commercially available genomics-based non-invasive prenatal tests that we compared include four WGS based tests [16], one test using analysis SNP [17], one test that utilizes microarray technology [18], and one test employing the novel rolling circle replication-based method [19].

### 2.2. Study Selection and Search Strategy

We collected data from studies published in six databases—PubMed, Embase, Web of Science, Scopus, ClinicalTrials.gov, and Cochrane library—over the past 20 years (January 2003 to October 2023). The following keywords were used: “Nifty trisomy” OR “Nifty Down syndrome” for the NIFTY test, “Genetech trisomy” OR “Genetech Down syndrome” for the GeneTech test, “Verifi trisomy” OR “Verifi Down syndrome” for the Verifi test, “PrenaTest” for the PrenaTest, “Panorama trisomy” OR “Panorama Down syndrome” for the Panorama test, “Harmony trisomy” OR “Harmony Down syndrome” for the Harmony prenatal test, and “Vanadis trisomy” OR “Vanadis Down syndrome” for the Vanadis test. For simplicity, we did not include trisomy 18 or 13 as search terms, as the commercially available NIPT tests already screen for all these aneupoidies. Language restrictions were not used. Commercial names had to be kept to ensure accurate database searches.

### 2.3. Conditions Being Studied

The study focuses on prenatal screening for fetal chromosomal abnormalities, specifically trisomies 21 (Down syndrome), 18, and 13, through non-invasive prenatal testing (NIPT). NIPT utilizes cell-free DNA (cfDNA) from maternal blood to screen for these abnormalities. The review compares the sensitivity, specificity, and efficacy of different methods of cfDNA detection, including whole-genome sequencing (WGS), and targeted techniques like single nucleotide polymorphisms (SNPs), microarray, and rolling circle amplification (RCA). The goal is to evaluate the clinical performance and utility of these methods in the early detection of common aneuploidies.

The inclusion criteria were studies on non-invasive prenatal testing (NIPT) utilizing each cfDNA for trisomy screening during human pregnancy, with available data on true positive (TP), false positive (FP), false negative (FN), and true negative (TN) counts.

The exclusion criteria were non-human studies, summaries, case reports, in vitro studies, studies combining multiple NIPT tests without differentiation, or studies lacking extractable data on TP, FP, FN and TN counts.

### 2.4. Data Extraction

The following characteristics were extracted from the included studies: authors, year of publication, brand name of the NIPT, techniques used in the NIPT, study design, conformation method, patient risk status prior to NIPT, gestational week at testing, sample size, target condition, test failure rate, percentage of aneuploid samples, percentage of T21, T18 and T13 samples and percentage of euploid samples. The details of each characteristic are explained below.

### 2.5. Exposure

The exposure is pregnant women undergoing non-invasive prenatal testing (NIPT).

Inclusion criteria: studies of the non-invasive prenatal test (NIPT) utilizing each cfDNA for trisomy screening during human pregnancy, with true positive (TP) count, false positive (FP) count, false negative (FN) count, and true negative (TN) count.

If mentioned within the article, sensitivity, specificity, positive predictive value (PPV), and negative predictive value (NPV) were also collected. For studies that did not report these values, they were calculated using the equations shown below.

The eligibility criteria for each of the reviewed articles were that each characteristic extracted from the studies must adhere to the following:

- Techniques of NIPT—The cfDNA screening methods of WGS or targeted techniques. Study design—Randomized studies (pregnant women were randomly assigned to receive a specific NIPT and the reference standard). Retrospective and prospective cohort studies (all pregnant women were tested with one or more NIPT method and the reference standard), as well as retrospective and prospective case–control studies (one or more NIPT methods were compared with the reference standard). Although retrospective or case–control studies are prone to bias, we included them due to the anticipated limited number of studies meeting the inclusion criteria. When sufficient data were available, sensitivity analyses were conducted to assess the impact of excluding case–control studies;
- Target conditions—We considered seven fetal aneuploidies, with a primary focus on T21, T18 and T13.
- Control/confirmation methods—Fetal karyotyping is performed on cells obtained from chorionic villus sampling (CVS), amniotic fluid, placental tissue, fetus lost due to miscarriage, or other equivalent and recognized methods performed on the same materials. We used the term “fetal karyotyping”, but it included traditional banding techniques, spectral karyotyping, fluorescence in situ hybridization (FISH), array comparative genomic hybridization (microarray), and quantitative fluorescence polymerase chain reaction (QF-PCR). If fetal karyotyping was not performed, neonatal clinical examination or medical records from birth were used as a secondary reference standard for T21, T18, or T13;
- Prior risk—Whether patients were tested with NIPT as a first or second screening after conventional screening for those classified as high-risk for fetal aneuploidies;
- Test week—The week of gestation at which patients were tested.
- Sample size—The number of participants in the studies. We included women of all ages, ethnicities and gestational ages with singleton or multifetal (monochorionic and dichorionic) pregnancies.

The equations of sensitivity, specificity, positive predictive value (PPV), and negative predictive value (NPV) are as follows [20]: sensitivity = TP/(TP + FN), specificity = TN/(TN + FP), PPV = TP/(TP + FP), and NPV = TN/(FN + TN). Here, TP stands for true positive, TN, for true negative, FP is false positive and FN is false negative.

The main outcome was to determine the sensitivity, specificity, PPV, NPV, TP, FP, FN and TN of NIPT used for detecting fetal chromosomal abnormalities with the different methods.

### 2.6. Statistics

We calculated sensitivity, specificity, PPV and NPV, as well as the 95% confidence index, for T21, T18 and T13 and for all publications [21,22]. To calculate the effect sizes for each NIPT, the R for Windows version 4.4.2 environment (R Foundation for Statistical Computing, 2024, Vienna, Austria) and the R package meta (version 8.0-2) were used. It has to be highlighted that although meta-analysis methods were used to calculate the effect sizes, as stated previously, the aim of this study was not to statistically compare the commercially available NIPT products; therefore, all *p*-values were omitted. Furthermore, the following issues were identified during the analysis, which also prevented the proper and direct comparison of the tests. (1) The subgroup sizes were low with a maximum of *n* = 5. (2) In the case of T13, there were some studies wherein the TP, FP and FN values were zero, which resulted in the need to exclude those studies. Although the underlying mathematical theories suggest that both specificity and PPV could be calculated using the limit of 0, no such algorithms are available currently. (3) In relation to the small subgroup sizes, some effect size calculations, particularly for their confidence intervals, were significantly affected by the lower TP sample sizes, which ultimately resulted in biased confidence intervals. The meta analysis see Supplementary Figures S1–S12 and Supplementary Tables S1–S3.

## 3. Results

### 3.1. Study Inclusion for the Systematic Review

**Figure 1:**
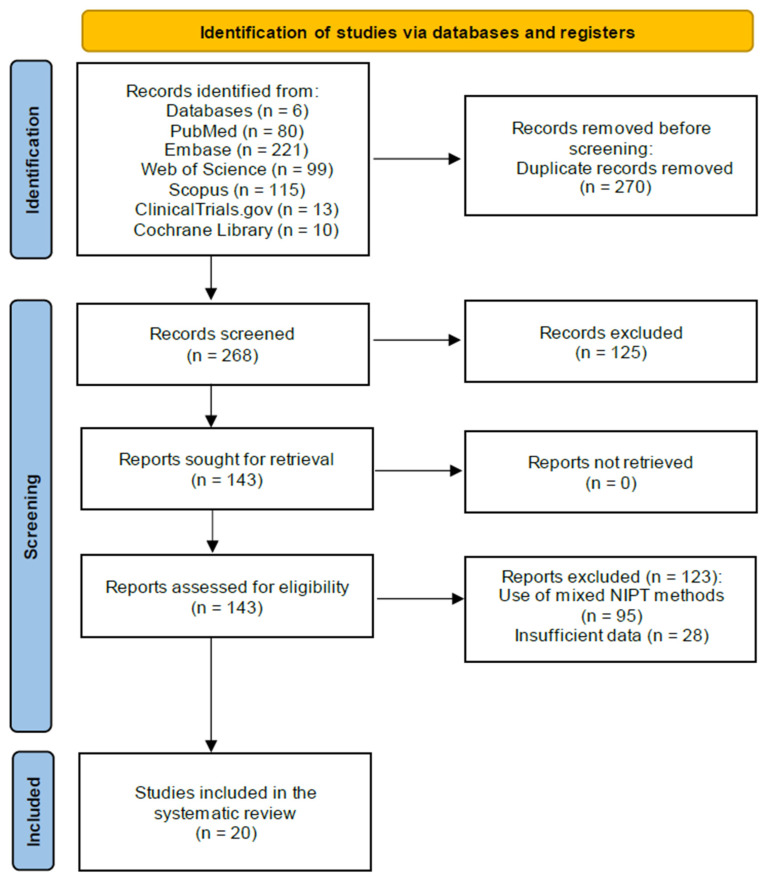
PRISMA study flow diagram for systematic reviews.

Our database search, conducted between January 2003 and October 2023, yielded a total of 538 articles across six databases (80 from PudMed, 221 from Embase, 99 from Web of Science, 115 from Scopus, 13 from ClinicalTrials.gov, and 10 from Cochrane Library). After removing 270 duplicates, two independent review authors screened the titles and abstracts of 268 publications, excluding 125 as irrelevant. Full-text reviews of the remaining 143 articles led to the exclusion of 95 due to the use of mixed NIPT methods and 28 due to insufficient data (lacking counts for true positives, false positives, true negatives, and false negatives). Ultimately, 20 articles (including 21 studies) involving 92,164 pregnant women (including 1245 T21 patients) met our inclusion criteria. These studies, published between 2012 and 2023, used WGS (10 studies), SNPs (2 studies), microarray (4 studies), and RCA (5 studies) (Figure 1: PRISMA study flow diagram for systematic review).

### 3.2. Basic Features of the Included Studies

The characteristics of the included studies are summarized in the table below (Table 1: Characteristics of included studies). The studies analysed various commercially available NIPT tests, as follows:

- WGS-based tests—Three studies on the first product [23,24,25], one study on the second product [26], and four studies on the third product [27,28,29,30], with two studies on the third test [31,32];
- SNP method—Two publications [17,33];
- Microarray method—Four studies [18,34,35,36];
- RCA method—Five studies [1,19,34,37,38].

Here, 13 articles were prospective cohort studies, 6 papers were retrospective cohort studies, 1 article was a cross-sectional study with data from prospective studies, and 1 paper concerned both prospective and retrospective studies.

**Table 1:** Characteristics of the included studies.

Study Title	Brand Name	Techniques	Study Design	Target Condition	Conformation Method	Prior Risk	Test Week	Sample Size	Failure Rate
Lau 2014 [24]	NIFTY	WGS	prospective cohort	T21, T18, T13, XXX, X, MM X/XX, M X/XY, XXY/XYY, AT, TT, Deletion/Duplication	CSV, AC, G-band, QF-PCR, aCGH	Any	After 12 week	1981	1.16
Jiang 2012 [23]			prospective cohort	T21, T18, T13, X, XXY/XYY,	CSV, AC, karyotype, FISH, QF-PCR	Unknown	10–34 week	903	Unknown
Van 2015 [25]			retrospective cohort	T21, T18, T13, X, XXY/XYY,	CSV, AC	Unknown	Unknown	683	1.46
Sasaki 2021 [26]	GeneTech		cross-sectional study with data from prospective cohort	T21, T18, T13	CSV, AC, POC, NK	Unknown	10–32 weeks	44,263	Unknown
Kershberg 2015 [29]	Verifi		prospective cohort	T21, T18, T13, M21, M18, X, XXY/XYY,	Unknown	High risk	Unknown	6608	Unknown
Marchili 2015 [30]			prospective cohort	T21, T18, T13, X,XXX	Unknown	Any	Unknown	614	Unknown
Horelli-Kuitunen 2019 [27]			prospective cohort	T21, T18, T16, T13, T9, M18, M13, X,XXX, XXY/XYY	CSV, AC	Any	Unknown	965	Unknown
Togneri 2019 [28]			215 retrospective and 840 prospective cohort	T21, T18, T13	AC, CSV, POC, NK, clinical	Any	9.1–37.3 week	1000	0.24
Stumm 2013 [32]	PrenaTest		retrospective cohort	T21, T18, T13	CSV, AC	Any	Unknown	517	Unknown
Hofmann 2014 [31]			prospective cohort	T21, T18, T13	Unknown	Any	Unknown	5600	Unknown
Bajka 2022 [17]	Panorama	SNP	retrospective cohort	T21, T18, T13, X, XXY/XYY, VTT, MD	CSV, AC	Any	After 9 week	7549	Unknown
Verma 2018 [33]			retrospective cohort	T21, T18, T13, SCA	CSV, AC, FISH, karyotyping	Any	11–18 week	499	Unknown
Conotte 2022 [34]	Harmony	Microarray	prospective cohort	T21, T18, T13	PIT	Unknowns	10–38 week	900	3.2
de Wergifosse 2021 [35]			retrospective cohort	T21, T18, T13	AC, NK	Any	After 10 week	3114	2.8
Pérez-Pedregosa 2014 [36]			prospective cohort	T21, T18, T13	PIT, NK	Unknown	After 10 week	582	2.9
Willems 2014 [18]			prospective cohort	T21, T18, T13	CSV, AC	Any	10–30 week	2968	0.9
Gormus 2021 [38]	Vanadis	RCA	retrospective cohort	T21, T18, T13	PIT, NK	Any	10–40 week	848	0.93
Palomaki 2022 [39]			prospective cohort	T21, T18, T13	karyotype, MA, clinical.	Any	10–20 week	2350	0.04
Conotte 2022 [34]			prospective cohort	T21, T18, T13	PIT	Unknown	10–38 week	900	0.2
Pooh 2021 [37]			prospective cohort	T21, T18, T13	CSV, AC	High risk	After 11 week	1208	Unknown
Saidel 2023 [19]			prospective cohort	T21, T18, T13	CSV, AC, US, clinical	Any	After 10 week	8112	0.07

Though the target conditions of each study varied between the articles, T21, T1 and T13 were included in all 20 studies. Other conditions covered by some of the studies included Monosomy 21, Monosomy 18, Monosomy 13, autosomal trisomy (AT), triple trisomy (TT), and sex chromosomal abnormalities such as 45X, 47XXX, Maternal mosaic X/XX (MM X/XX), Mosaic X/XY (M X/XY), 47XXY/XYY, Deletion/Duplication, triploidy or vanishing twins (VTT) and different microdeletions (MD).

Each study employed a variety of confirmational methods, most commonly prenatal invasive tests such as chorionic villi sampling (CSV) and/or amniocentesis (AC). In some cases, the product of conception was sampled, while in a minority of the cases, neonatal karyotyping (NK) was used to confirm the prenatal diagnosis. The confirmatory test in individual cases also included fluorescence in situ hybridization (FISH), conventional G-banding cytogenetic studies, quantitative fluorescent polymerase chain reaction (QF-PCR) and array comparative genomic hybridization (microarray).

In two studies, samples were collected from patients with high risk of genetic abnormalities, such as maternal age over 35 years, a positive family history of genetic abnormalities, a history of genetic disorders in a previous pregnancy, maternal disease, and a positive result from primary screening using ultrasound or biochemical markers. Eleven studies included low-risk patients who underwent NIPT. Eight articles did not specify the risk status of participants prior to NIPT.

The range of gestational weeks during which each study was conducted also varied. Since NIPT is typically performed any time after the 10th gestational week, most studies included results from samples collected within this timeframe.

The sample sizes of the studies also varied widely. Ten studies included fewer than 1000 participants, six studies collected samples from 1000 to 5000 patients, four studies analyzed data from 5000 to 10,000 individuals, and one study included 44,263 samples. Some papers did not provide details about patient selection or specify inclusion and exclusion criteria. We also extracted the failure rates of the tests; however, the reported results varied significantly, and eight studies did not mention this.

### 3.3. Findings

A total of 21 studies assessing NIPT from 2012 to 2023 detected 1245 T21-affected and 90,919 non-T21-affected pregnancies. Two studies enrolled pregnant women with a high risk of fetal aneuploidy, and eleven studies enrolled women with various prior risks, including no risk (mixed risk). Eight studies did not specify the prior risk of participants. Of 21 studies, the numbers of papers involving WGS, SNPs, microarray and RCA methods were 10, 2, 4, and 5, respectively.

The results are summarized in Table 1.

The following data was extracted from each study: sample size, true positive (TP) count, false positive (FP) count, false negative (FN) count, true negative (TN) count and, where available, sensitivity, specificity, positive predictive (PPV), and negative predictive values (NPV). If these values were not included, we calculated them using the equations shown previously (see data extraction for details).

The total sample sizes of the four WGS-based tests, the SNP-based test, the microarray-based test, and the RCA-based test were 3567, 44,263, 9187, 6117, 8048, 7564, and 13,418, respectively. The smaller sample sizes observed in studies on the first WGS-based test may impact the reliability of results across different NIPT methods.

## 4. Discussion

### 4.1. Summary of Main Results

This review includes data from 21 studies involving 92,164 pregnant women, of whom 1245 were affected by T21, tested using genomics-based non-invasive prenatal testing (NIPT).

Out of the included studies, eleven were conducted in unselected populations, two in high-risk cohorts, and eight studies did not specify how the participants were recruited. In the two studies involving high-risk participants, two NIPT methods were evaluated—the WGS method [29] and the RCA method [37]. Both studies showed high accuracy in detecting the three major trisomies (T21, T18 and T13), with sensitivities ranging from 95.0% to 100% depending on the trisomy, and specificity above 99%.

Because of the different sample sizes, we calculated the weighted average for the different NIPT methods. This was done by multiplying each data point by its sample size, summing the products, and then dividing it by the total number of samples.

We compared the averages of sensitivity, specificity, PPV, and NPV for each NIPT method used for T21 detection (Table 2 and Table 3). Values lower than 95% and higher than 90% were colored blue, while values lower than 90% were highlighted red. As for sensitivity, the RCA test showed slightly lower values (97.63%), while all the other tests, such as the WGS-based tests, the SNP test, and the microarray test, showed high sensitivity (above 98.9%). This difference might be the result of the slight variations and limited number of studies. Therefore, it cannot be concluded that the RCA method has significantly lower sensitivity than the other methods. In contrast, all tests showed similarly high specificity values exceeding 99%.

Regarding the positive predictive value, NIPT cannot reach 100%, as the cfDNA used is of placental origin, and may harbor confined placental mosaicism. Further, 100% PPV was not reached for T21 as the SNP and RCA tests had 92.97% and 75.98% PPVs, respectively. Two publications were outliers, with PPVs of 80.0% in one of the SNP tests [33] and 64.44% in one the RCA tests reported [19], partly explaining the relatively lower PPVs for these tests. It is worth noting that when we excluded these two studies, the average PPVs of the SNP and RCA tests still remained lower than that of other tests (93.83% and 93.25%), supporting the previous result that the SNP and RCA tests have lower PPVs for T21 detection. In terms of NPV, all NIPT methods performed well, with values above 99%.

In conclusion, the figures show that all tests utilizing WGS or targeted methodologies such as SNPs, microarrays, and RCA perform similarly in terms of clinical sensitivity, specificity and NPV for the detection of fetal T21. However, two targeted methods—using SNPs and RCA—demonstrated lower PPV values compared to other NIPT methods.

When we compared the weighted averages of sensitivity, specificity, PPV and NPV of each NIPT method used for detecting T18 (Table 4 and Table 5), the microarray-based test showed lower sensitivity, at 77.22%. This was primarily due to the low sensitivity scores of all but one report on the microarray-based methods, with sensitivities of 73.33%, 71.43%, 100%, and 80%, respectively [18,34,35,36]. Based on these results, the microarray-based (Harmony) test showed lower sensitivity for the detection of T18. The WGS-based methods performed well in terms of sensitivity, nearing 100%. One product showed slightly lower sensitivity, at 96.40%, which can be explained by one outlier in a single study reporting 95% sensitivity, while the rest of the tests reported 100% sensitivity [27,28,29,30]. For this reason, we cannot conclude that this particular WGS-using product has lower sensitivity than the other tests. All NIPT tests proved to be highly specific for detecting T18, with specificity above 99%, regardless of the method or product. In terms of PPV, the values varied significantly, with two WGS tests scoring only 77.56% and 84.74%, and the RCA test scored only 66.98%. One of the WGS products did particularly poorly, with PPV for T18 ranging between 66.67% and 83.33% [27,28,29,30].

Interestingly, when we looked more closely at the performances of the other WGS-based test, the PPV for T18 was 100% and 77.27% [31,32]. But the sample size for the Stumm study [32] was approximately one-tenth of that in the Hofmann study [31], suggesting that the PPV for this product is clearly lower than those of other methods. Based on the published figures, the PPV of T18 detection using the RCA test was lower, with the two lowest scores—60% and 65.12%—associated with the studies with the largest sample (Table 4) [19,34,37,38,39]. While two of the WGS-based methods and the RCA test had lower PPVs for T18 detection, another WGS-based NIPT had only a slightly lower PPV of 92.56% based on one study, but with a relatively high number of tested cases. There was no significant difference for the PPV between one of the WGS tests, the SNP, and the microarray-based test (98.05%, 95.31%, and 100%, respectively). Each NIPT method had a high NPV (all above 99%).

In summary, all types of non-invasive prenatal tests, utilizing WGS, SNPs, microarray or RCA, perform similarly in terms of clinical specificity and NPV for the detection of fetal T18. The microarray method, however, had lower sensitivity compared to other tests. All tests demonstrated relatively lower PPV, except for one test utilizing WGS, as well as the microarray-based method.

In terms of sensitivity, there was only one outlier in the RCA group [39], with 62.5% sensitivity, compared to all the other tests, which had 100% sensitivity (Table 6). All tests are found to be similarly efficient when comparing the weighted averages of sensitivity, specificity, PPV and NPV of each NIPT method for T13 detection (Table 7). For specificity, all NIPT methods performed well, with values above 99%, thus we did not detect any differences in specificity between the tests. There was a relatively high PPV in all tests, at above 99%, except for one of the WGS methods, which had a PPV of only 84.74%. Similarly to the PPV in T18, the same authors reported a low PPV figure for T13 testing in a large study population, which lowered the overall PPV for this product (100% and 83.33%, respectively) [31,32]. Every NIPT method had a high NPV, above 99%, for T13.

As a conclusion, these results show that nearly all tests utilizing whole-genome sequencing (WGS) or targeted methods such as single nucleotide polymorphisms (SNPs), microarrays, and rolling circle amplification (RCA) perform similarly in terms of clinical sensitivity, specificity, PPV, and NPV for the detection of fetal T13.

### 4.2. Comparison with Classic Screening Methods and Health Economical Considerations

In the 21 selected studies, all NIPT methods showed greater sensitivity for the detection of T21, above 98%, compared to traditional screening tests; the detection rate of the combined test is 82–87%, while NT alone could detect 70% of Down syndrome (DS) cases. The detection rate of the quadruple test is 81%, and the detection rates of the full integrated test, serum integrated test, and stepwise sequential testing are 96%, 88% and 95%, respectively [13].

It is not our aim to “compare” the traditional methods with the new cfDNA-based technique. Nevertheless, in the recent era, considerations of cost-effectiveness are becoming increasingly important, and the issue is far more complex than simply comparing the cost of the tests themselves. In the past, the use of NIPT as an alternative to current first-tier screening tests, which include both biochemical and ultrasound methods, has been deemed non-cost-ineffective [10,40].

Changes in the cost of NIPT have led to its adoption as a first-tier screening test in several countries [41,42].

Looking at long-term health and economic considerations, it transpired that including NIPT in existing prenatal screening for DS was beneficial over conventional testing [43]. The authors also stated that, based on the prices at the time, using NIPT as a second-tier screening method is more cost-effective than NIPT as the first choice. But long term costs were considered by Xiao et al., who compared the costs of different diagnostic strategies for Down syndrome in a large population (17,363 patients). Down syndrome seems to have the largest impact on society, since it is compatible with life; the overall cost also includes long-term direct medical and indirect, non-medical health and social costs, such as the development of services and education for individuals with Down syndrome. They observed three groups: The first group used classical serum screening, and subsequent invasive testing if the Down syndrome risk was ≥1/270. The second group employed serum screening, and those with a T21 risk ≥ 1/270 underwent invasive testing, while those with a risk between 1/270 and 1/1000 had NIPT, followed by invasive testing based on the NIPT result. The third method applied NIPT to all patients with a risk ≥ 1/1000, and if indicated, this was followed by invasive tests. In the fourth method, all patients underwent NIPT [44]. In summary, serum screening alone would have missed 14 cases of Down syndrome, while the 2nd and 3rd approaches would have resulted in eight missed cases, and none would have resulted with the fourth method (NIPT for all). When they compared the lifetime health economic costs of the different strategies, group 1 was the most expensive and resulted in the most “missed” Down syndrome cases. The “lifetime” costs, for groups 1, 2, 3 and 4 were 100%, 63%, 61%, and 23%, respectively.

A recent publication concluded that the application of ultrasound combined with NIPT in prenatal testing as a complimentary method seems to be the most effective approach, providing the most accurate data for obstetricians and resulting in the best health and economic outcomes [45].

We would like to conclude our considerations with the 2023 consensus statement of the board of the International Society for Prenatal Diagnosis on the use of non-invasive prenatal testing for the detection of fetal chromosomal conditions in singleton pregnancies, as follows: [46] (1) NIPT is the most accurate screening test for common autosomal aneuploidies (trisomies 21, 13 and 18) in unselected singleton populations, and those at known increased probability. (2) False positive results occur with NIPT. Therefore, ISPD strongly recommends that all pregnant individuals with a high chance of an NIPT result undergo genetic counseling and diagnostic testing if they are considering termination of pregnancy.

### 4.3. Comparison with Other Systematic Reviews and with Meta Analysis

In these 20 studies on the common trisomies, we did not find any specific differences in accuracy between the detections of T21, T18 and T13. On the other hand, one published systematic review of NIPT stated that targeted massively parallel sequencing (TMPS) appeared to be accurate for the detection of T21, with lower accuracy for T18 and T13 [8].

### 4.4. Strengths and Limitations

#### 4.4.1. Strengths

We did not find any other reviews focusing on the differences in accuracy between the different NIPT methods for common aneuploidies, such as WGS, or targeted methodologies with SNPs, microarray, and RCA. Therefore, this review offers a unique perspective on the efficacy of using NIPT for common trisomy detection, providing valuable insights for individuals when selecting commercially available NIPT brands.

This review includes data from 92,164 pregnant women, a relatively large sample size, and focuses on seven popular commercially available brands.

#### 4.4.2. Limitations

The sensitivity and specificity reported in these studies are not of optimal quality due to the small number of patients enrolled in some studies for the different NIPT methods, the unequal sample sizes across studies, and the varying conditions under which the samples were collected. Additionally, due to the small number of studies, it was not possible to assess publication bias or outliers. However, the risk of bias was generally high in terms of patient selection and timing. Some women may experience spontaneous pregnancy loss after being enrolled in a study. However, none of the studies reported such events, and in most cases, genetic tests were not performed to confirm aneuploidy. Since women with spontaneous abortions are unlikely to undergo follow-up, we believe that any potential risk of bias has been accounted for in the quality assessment of the studies. We acknowledge that the studied papers cover a heterogenous patient population that generates a degree of bias as the PPV is higher in high-risk studies.

### 4.5. Applicability of Findings to the Review Question

These results imply that NIPT is a highly sensitive and specific method for identifying fetal aneuploidies. The majority of the research focused either on high-risk pregnancies or mixed populations, in which it was impossible to distinguish between pregnant women who were not selected and those with high-risk pregnancies. Therefore, more research on pregnant women in general is required before definitive conclusions regarding the sensitivity of NIPT as a first-tier screening test can be drawn. The performances of the four different NIPT method types (WGS, SNP, microarray, and RCA) seem to be comparable, but there are many more NIPT assays available for each approach than were compared in this review. There is a dearth of published data regarding the diagnostic accuracy of these methods. Furthermore, due to various factors, such as variations in gestational age or mean body mass index, performance within the cohorts under investigation may not be indicative of performance within other populations. Crucially, the probabilities connected to any given patient sample may differ significantly from the summary sensitivities and specificities obtained from cohort data. Lastly, there was a high risk of bias and generally poor methodological quality in the studies, particularly with regard to patient selection, flow, and timing. Therefore, it is not possible to use each method’s summarized sensitivity, specificity, and related predictive values as direct indicators of the likelihood that a specific patient’s sample will be affected by a positive or negative result [8].

### 4.6. Further Considerations

In the past, the use of NIPT as an alternative to current first-tier screening tests, which include both biochemical and ultrasound methods, was deemed cost-ineffective [10,47]. More recently, a number of countries have adopted cfDNA-based NIPT as the single first-tier screening method for trisomies [41].

It is worth noting that, compared to targeted methods such as SNPs, microarrays, and RCA, WGS have the potential to screen for a variety of chromosomal abnormalities. There is insufficient data on the clinical sensitivity of detecting rare abnormalities, and it is beyond the scope of this paper to comment on microdeletion detection or single-gene NIPT. It is not documented in this study, but needs to be mentioned, that triploidy can only be diagnosed with the SNP method [48,49]. WGS benefits only a very small number of patients who actually have rare abnormalities, but it also increases false positive rates, identifying patients as high-risk pregnancies, resulting in heightened surveillance and unnecessary invasive diagnostics. Currently, WGS is more expensive than other targeted methods such as SNPs, microarrays, or RCA. More data are required, and financial feasibility issues should be addressed in order to prove the potential of WGS-based whole-chromosome screening [50,51,52].

We did not specifically address the failure rates of NIPT. Gil et al. (2017) [53], in their metanalysis, addressed this problem, referring to the “no result” of cfDNA-based tests. The three reasons they quote are as follows: Problems with blood collection and the transportation of the sample, and issues with hemolysis, etc. (0.03–11.1%). The second issue is the low fetal fraction, below 4% (0.1–6.1%), and the third reason is assay failure (failed DNA extraction, amplification or sequencing). They also state that it was not possible to draw a correlation between the failure rates and the method used for the analysis, but it seemed that the failure rate for sex chromosome aneuploidies was higher than for trisomies [53]. A look at the failure rate of the different tests gave the following result: for the WGS-based method, failure rates ranged from 0.24% to 1.46%; with the microarray, the highest failure rate was 3.2% (0.04–3.2%), whilst the RCA failure rate ranged from 0.07 to 0.93%. Considering the above-mentioned reasons for the “no result”, comparing failure rates seems unreliable due to the heterogeneity of the methods and the bias caused by human factors.

With regard to the versatility, WGS has the most benefits. Apart from trisomies, it can detect sex chromosome abnormalities, and also microdeletions. Its technology requires a simpler and faster laboratory pipeline. Targeted methods, such as the SNP and microarray, are similarly adaptable. SNP requires the multiplex amplification of SNP sequencing in a single PCR reaction, followed by next-generation sequencing. SNP can detect common aneuploidies, sex chromosome alterations, microdeletions and also monogenic diseases. Microarray-based technology is currently available for the detection of common trisomies, sex chromosome aneuploidy and DiGeorge microdeletion. RCA, which is an easy test, will give fewer answers (only trisomies), but the method offers a simpler and more cost-effective pipeline [54].

## 5. Conclusions

In conclusion, for the detection of fetal Down syndrome, these results show that whole-genome sequencing (WGS), and targeted methods such as single nucleotide polymorphisms (SNPs), microarrays, and rolling circle amplification (RCA), perform similarly in terms of clinical sensitivity, specificity and NPV, but the targeted methods using SNPs, and RCA had lower PPVs compared to other NIPT methods.

For the detection of fetal T18, WGS, SNP, microarrays, and RCA perform similarly in terms of clinical specificity and NPV. The targeted method using the microarray had lower sensitivity compared to other methods. In terms of PPV, one of the products utilizing WGS, and the targeted method using RCA showed lower values.

All the tests, utilizing WGS, SNPs, microarrays, and RCA, performed similarly in terms of clinical sensitivity, specificity, PPV, and NPV for the detection of fetal T13.

We also compared the accuracy of each NIPT method for all three trisomies—T21, T18 and T13. Under this approach, there was no significant difference in diagnostic accuracy between the different trisomies. All methods showed high utility in the clinical setting with accurate results, and there was no significant difference between the examined NIPT methods considering common aneuploidies. When the scope of screening is different, WGS-based tests have much wider potential.

Our review gives a robust overview and comparison of the efficacy of NIPT for detecting common trisomies, comparing the efficacy of seven popular commercial brands utilizing four different methods of NIPT. This review is unique in offering clinicians independent advice when choosing between commercially available NIPT brands.

## Figures and Tables

**Table 2:** Accuracy of NIPT tests for trisomy 21.

T21	Study Title	Sample Size	TP	FP	FN	TN	Sensitivity	Specificity	PPV	NPV
NIFTY	Lau 2014 [24]	1981	23	0	0	1958	100.00	100.00	100	100.00
	Jiang 2012 [23]	903	16	0	0	887	100.00	100.00	100	100.00
	Van 2015 [25]	683	2	0	0	681	100.00	100.00	100	100.00
GeneTech	Sasaki 2021 [26]	44,263	459	13	1	43,790	99.78	99.97	97.25	100.00
Verifi	Kershberg 2015 [29]	6608	104	6	1	6497	99.05	99.91	94.55	99.98
	Marchili 2015 [30]	614	12	0	0	602	100.00	100.00	100	100.00
	Horelli-Kuitunen 2019 [27]	965	27	0	0	938	100.00	100.00	100	100.00
	Togneri 2019 [28]	1000	47	0	1	952	97.92	100.00	100	99.90
PrenaTest	Stumm 2013 [32]	517	39	0	1	477	97.50	100.00	100	99.79
	Hofmann 2014 [31]	5600	82	2	0	5516	100.00	99.96	97.62	100.00
Panorama	Bajka 2022 [17]	7549	76	5	0	7468	100.00	99.93	93.83	100.00
	Verma 2018 [33]	499	8	2	0	489	100.00	99.59	80	100.00
Harmony	Conotte 2022 [34]	900	34	0	1	865	97.14	100.00	100	99.88
	de Wergifosse 2021 [35]	3114	17	0		3097	100.00	100.00	100	100.00
	Pérez-Pedregosa 2014 [36]	582	14	0	0	568	100.00	100.00	100	100.00
	Willems 2014 [18]	2968	51	0	1	2916	98.08	100.00	100	99.97
Vanadis	Gormus 2021 [38]	848	19	2	0	827	100.00	99.76	90.48	100.00
	Palomaki 2022 [39]	2350	84	5	1	2260	98.82	99.78	94.38	99.96
	Conotte 2022 [34]	900	35	2	0	863	100,00	99.77	94.59	100.00
	Pooh 2021 [37]	1208	58	4	1	1145	98.31	99.65	93.55	99.91
	Saidel 2023 [19]	8112	29	16	1	8066	96.67	99.80	64.44	99.99

**Table 3:** Weighted average values for T21.

T21	Sensitivity	Specificity	PPV	NPV
NIFTY	100.00	100.00	100.00	100.00
GeneTech	99.78	99.97	97.25	100.00
Verifi	99.09	99.93	96.08	99.98
PrenaTest	99.79	99.97	97.82	99.98
Panorama	100.00	99.91	92.97	100.00
Harmony	98.91	100.00	100.00	99.97
Vanadis	97.63	99.78	75.98	99.98

**Table 4:** Accuracy of NIPT tests for trisomy 18.

T18	Study Title	Sample Size	TP	FP	FN	TN	Sensitivity	Specificity	PPV
NIFTY	Lau 2014 [24]	1981	4	0	0	1977	100.00	100.00	100.00
	Jiang 2012 [23]	903	12	1	0	890	100.00	99.89	92.31
	Van 2015 [25]	683	1	0	0	682	100.00	100.00	100.00
GeneTech	Sasaki 2021 [26]	44,263	224	18	2	44,019	99.12	99.96	92.56
Verifi	Kershberg 2015 [29]	6608	38	11	2	6557	95.00	99.83	77.55
	Marchili 2015 [30]	614	2	1	0	611	100.00	99.84	66.67
	Horelli-Kuitunen 2019 [27]	965	11	3	0	951	100.00	99.69	78.57
	Togneri 2019 [28]	1000	5	1	0	994	100.00	99.90	83.33
PrenaTest	Stumm 2013 [32]	517	8	0	0	509	100.00	100.00	100.00
	Hofmann 2014 [31]	5600	17	5	0	5578	100.00	99.91	77.27
Panorama	Bajka 2022 [17]	7549	19	1	0	7529	100.00	99.99	95.00
	Verma 2018 [33]	499	1	0	0	498	100.00	100.00	100.00
Harmony	Conotte 2022 [34]	900	11	0	4	885	73.33	100.00	100.00
	de Wergifosse 2021 [35]	3114	5	0	2	3107	71.43	100.00	100.00
	Pérez-Pedregosa 2014 [36]	582	3	0	0	579	100.00	100.00	100.00
	Willems 2014 [18]	2968	4	0	1	2963	80.00	100.00	100.00
Vanadis	Gormus 2021 [38]	848	11	4	0	833	100.00	99.52	73.33
	Palomaki 2022 [39]	2350	28	15	1	2306	96.55	99.35	65.12
	Conotte 2022 [34]	900	14	0	1	885	93.33	100.00	100.00
	Pooh 2021 [37]	1208	46	6	0	1156	100.00	99.48	88.46
	Saidel 2023 [19]	8112	12	8	0	8092	100.00	99.90	60.00

**Table 5:** Weighted average value for T18.

T18	Sensitivity	Specificity	PPV	NPV
NIFTY	100.00	99.97	98.05	100.00
GeneTech	99.12	99.96	92.56	100.00
Verifi	96.40	99.82	77.56	99.98
PrenaTest	100.00	99.98	84.74	100.00
Panorama	100.00	99.99	95.31	100.00
Harmony	77.22	100.00	100.00	99.91
Vanadis	98.95	99.75	66.98	99.98

**Table 6:** Accuracy of NIPT tests for trisomy 13.

T13	Study Title	Sample Size	TP	FP	FN	TN	Sensitivity	Specificity	PPV
NIFTY	Lau 2014 [24]	1981	2	0	0	1979	100.00	100.00	99.90
	Jiang 2012 [23]	903	2	0	0	901	100.00	100.00	99.78
	Van 2015 [25]	683	0	0	0	683		100.00	100.00
GeneTech	Sasaki 2021 [26]	44,263	29	28	0	44,206	100.00	99.94	99.93
Verifi	Kershberg 2015 [29]	6608	15	6	0	6587	100.00	99.91	99.77
	Marchili 2015 [30]	614	0	0	0	614		100.00	100.00
	Horelli-Kuitunen 2019 [27]	965	3	0	0	962	100.00	100.00	99.69
	Togneri 2019 [28]	1000	12	2	0	986	100.00	99.80	98.81
PrenaTest	Stumm 2013 [32]	517	5	0	0	512	100.00	100.00	100.00
	Hofmann 2014 [31]	5600	5	1	0	5594	100.00	99.98	83.33
Panorama	Bajka 2022 [17]	7549	15	3	0	7531	100.00	99.96	99.80
	Verma 2018 [33]	499	1	0	0	498	100.00	100.00	99.80
Harmony	Conotte 2022 [34]	900	3	3	0	894	100.00	99.67	99.67
	de Wergifosse 2021 [35]	3114	1	4	0	3109	100.00	99.87	99.97
	Pérez-Pedregosa 2014 [36]	582	0	0	0	582		100.00	100.00
	Willems 2014 [18]	2968	2	0	0	2966	100.00	100.00	99.93
Vanadis	Gormus 2021 [38]	848	9	2	0	837	100.00	99.76	98.95
	Palomaki 2022 [39]	2350	5	2	3	2340	62.50	99.91	99.79
	Conotte 2022 [34]	900	3	1	0	896	100.00	99.89	99.67
	Pooh 2021 [37]	1208	12	0	0	1196	100.00	100.00	99.02
	Saidel 2023 [19]	8112	2	8	0	8102	100.00	99.90	99.98

**Table 7:** Weighted average values for T13.

T13	Sensitivity	Specificity	PPV	NPV
NIFTY	100.00	100.00	99.89	100.00
GeneTech	100.00	99.94	99.93	100.00
Verifi	100.00	99.91	99.68	100.00
PrenaTest	100.00	99.98	84.74	100.00
Panorama	100.00	99.96	99.80	100.00
Harmony	100.00	99.91	99.92	100.00
Vanadis	93.43	99.90	99.77	99.98

## Supplementary Materials

The following supporting information can be downloaded at: https://www.mdpi.com/article/10.3390/jcm14082813/s1, Figure S1: Specificity of the different tests in T21; Figure S2: Sensitivity of the different tests in T21; Figure S3: PPV of the different tests in T21; Figure S4: NPV of the different tests in T21; Figure S5: Specificity of the different tests in T18; Figure S6: Sensitivity of the different tests in T18; Figure S7: PPV of the different tests in T18; Figure S8: NPV of the different tests in T18; Figure S9: Specificity of the different tests in T13; Figure S10: Sensitivity of the different tests in T13; Figure S11: PPV of the different tests in T13; Figure S12: NPV of the different tests in T13; Table S1: Metaanalysis data for T21; Table S2: Metaanalysis data for T18; Table S3: Metaanalysis data for T13.

## Abbreviations

AFP	alpha-fetoprotein
cfDNA	cell-free DNA
CVS	chorionic villus sampling
FISH	fluorescence in situ hybridization
FN	false negative
FP	false positive
hCG	human chorionic gonadotropin
NGS	next-generation sequencing
NIPT	non-invasive prenatal test
NK	neonatal karyotyping
NPV	negative predictive value
NT	nuchal translucency
PIV	prenatal invasive test
PPV	positive predictive value
QF-PCR	quantitative fluorescence polymerase chain reaction
RCA	rolling circle amplification
SCA	sex chromosome aneuploidies
SNPs	single nucleotide polymorphisms
T13	Trisomy13
T18	Trisomy18
T21	Trisomy21
TN	true negative
TP	true positive
WGS	whole-genome sequencing
