# Supplementary material for: Systematic Review of Accuracy Differences in NIPT Methods for Common Aneuploidy Screening
Source: J Clin Med. 2025 Apr 18;14(8):2813. doi: 10.3390/jcm14082813 (PMC12028023; doi:10.3390/jcm14082813)
Supplement: Supplementary file 1 [file jcm-14-02813-s001.zip › jcm-3474712-supplementary.pdf]

Study	TN	TN + FP	Specificity	95% CI
**GeneTech**				
Sakaki 2021	43790	43803	0.9997	[0.9995; 0.9998]
**Harmony**				
Conotte 2022	865	865	1.0000	[0.9957; 1.0000]
Wergifosse 2021	3097	3097	1.0000	[0.9988; 1.0000]
Perez 2014	568	568	1.0000	[0.9935; 1.0000]
Willems 2014	2916	2916	1.0000	[0.9987; 1.0000]
**Group estimate:**		**7446**	**1.0000**	**[0.0000; 1.0000]**
**NIFTY**				
Lau 2014	1958	1958	1.0000	[0.9981; 1.0000]
Jiang 2012	887	887	1.0000	[0.9958; 1.0000]
Van 2015	681	681	1.0000	[0.9946; 1.0000]
**Group estimate:**		**3526**	**1.0000**	**[0.0000; 1.0000]**
**Panorama**				
Bajka 2022	7468	7473	0.9993	[0.9984; 0.9998]
Verma 2015	489	491	0.9959	[0.9854; 0.9995]
**Group estimate:**		**7964**	**0.9991**	**[0.9982; 0.9996]**
**PrenaTest**				
Stumm 2013	477	477	1.0000	[0.9923; 1.0000]
Hofmann 2014	5516	5518	0.9996	[0.9987; 1.0000]
**Group estimate:**		**5995**	**0.9997**	**[0.9987; 0.9999]**
**Vanadis**				
Gormus 2021	827	829	0.9976	[0.9913; 0.9997]
Palomaki 2022	2260	2265	0.9978	[0.9949; 0.9993]
Conotte 2022	863	865	0.9977	[0.9917; 0.9997]
Pooh 2021	1145	1149	0.9965	[0.9911; 0.9991]
Saidel 2023	8066	8082	0.9980	[0.9968; 0.9989]
**Group estimate:**		**13190**	**0.9978**	**[0.9968; 0.9985]**
**Verifi**				
Kershberg 2015	6497	6503	0.9991	[0.9980; 0.9997]
Marchili 2015	602	602	1.0000	[0.9939; 1.0000]
Kuitunen 2016	938	938	1.0000	[0.9961; 1.0000]
Togneri 2019	952	952	1.0000	[0.9961; 1.0000]
**Group estimate:**		**8995**	**0.9993**	**[0.9985; 0.9997]**

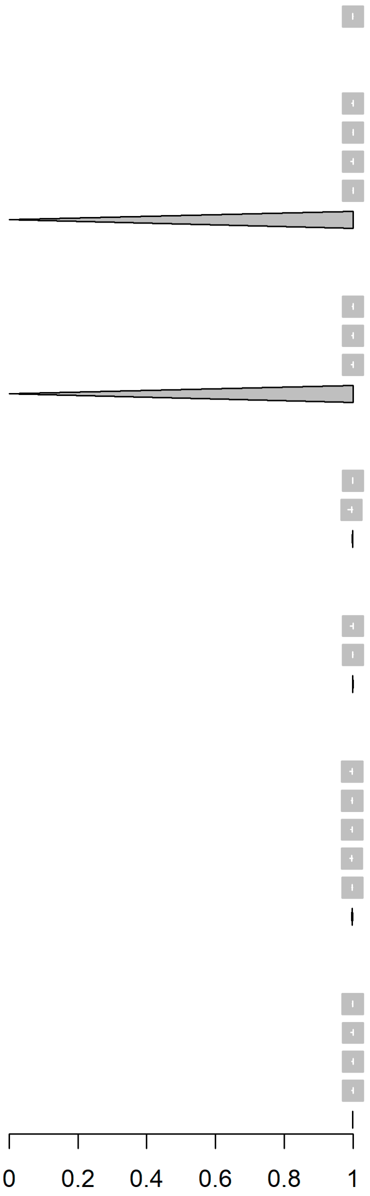

Supplementary Figure S1. Specificity of the different tests in T21.

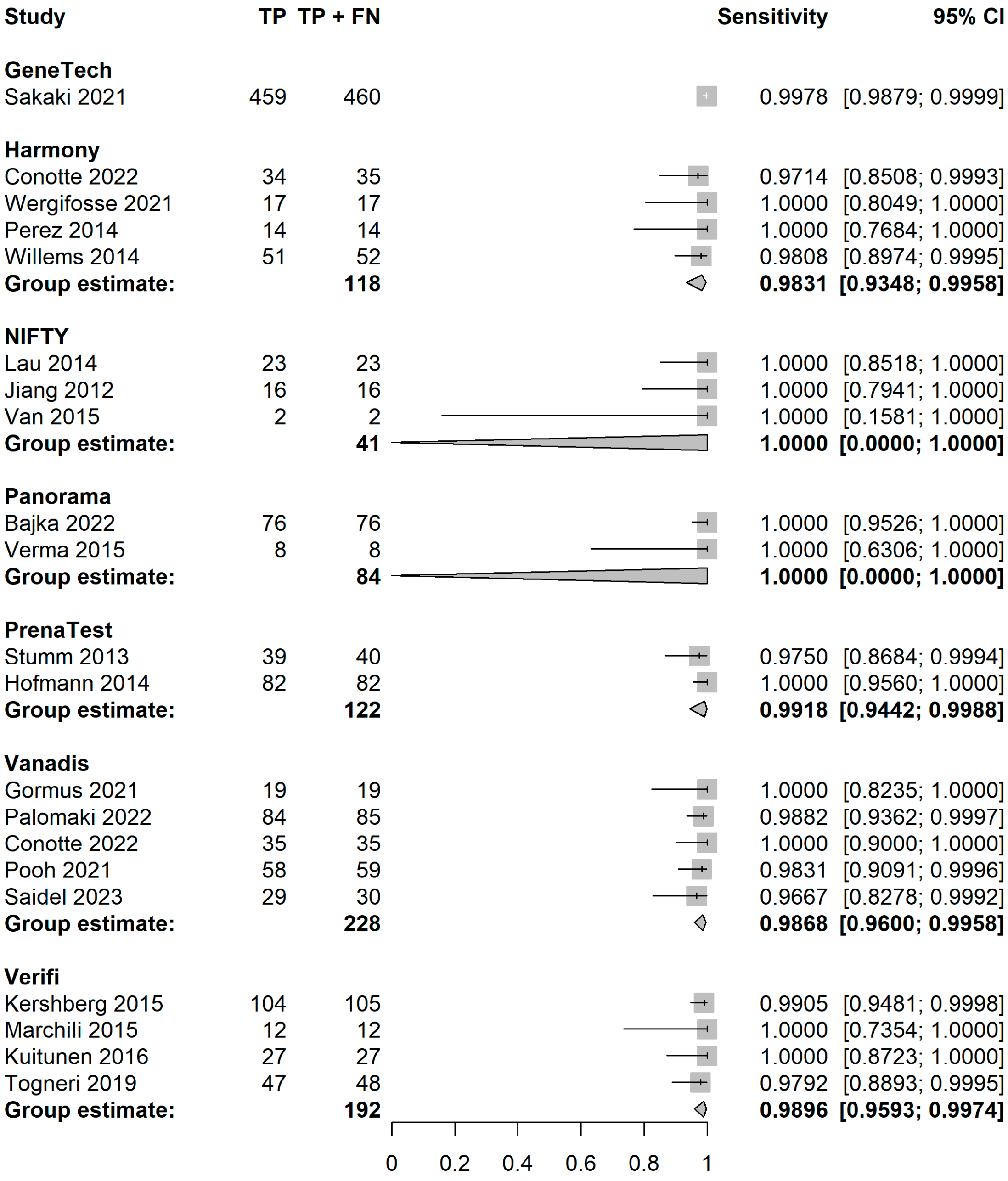

Supplementary Figure S2. Sensitivity of the different tests in T21.

Study	TP	TP + FP	PPV	95% CI
**GeneTech**				
Sakaki 2021	459	472	0.9725	[0.9534; 0.9853]
**Harmony**				
Conotte 2022	34	34	1.0000	[0.8972; 1.0000]
Wergifosse 2021	17	17	1.0000	[0.8049; 1.0000]
Perez 2014	14	14	1.0000	[0.7684; 1.0000]
Willems 2014	51	51	1.0000	[0.9302; 1.0000]
**Group estimate:**		**116**	**1.0000**	**[0.0000; 1.0000]**
**NIFTY**				
Lau 2014	23	23	1.0000	[0.8518; 1.0000]
Jiang 2012	16	16	1.0000	[0.7941; 1.0000]
Van 2015	2	2	1.0000	[0.1581; 1.0000]
**Group estimate:**		**41**	**1.0000**	**[0.0000; 1.0000]**
**Panorama**				
Bajka 2022	76	81	0.9383	[0.8618; 0.9797]
Verma 2015	8	10	0.8000	[0.4439; 0.9748]
**Group estimate:**		**91**	**0.9231**	**[0.8473; 0.9629]**
**PrenaTest**				
Stumm 2013	39	39	1.0000	[0.9097; 1.0000]
Hofmann 2014	82	84	0.9762	[0.9166; 0.9971]
**Group estimate:**		**123**	**0.9837**	**[0.9373; 0.9959]**
**Vanadis**				
Gormus 2021	19	21	0.9048	[0.6962; 0.9883]
Palomaki 2022	84	89	0.9438	[0.8737; 0.9815]
Conotte 2022	35	37	0.9459	[0.8181; 0.9934]
Pooh 2021	58	62	0.9355	[0.8430; 0.9821]
Saidel 2023	29	45	0.6444	[0.4878; 0.7813]
**Group estimate:**		**254**	**0.9035**	**[0.7918; 0.9584]**
**Verifi**				
Kershberg 2015	104	110	0.9455	[0.8851; 0.9797]
Marchili 2015	12	12	1.0000	[0.7354; 1.0000]
Kuitunen 2016	27	27	1.0000	[0.8723; 1.0000]
Togneri 2019	47	47	1.0000	[0.9245; 1.0000]
**Group estimate:**		**196**	**0.9892**	**[0.7004; 0.9997]**

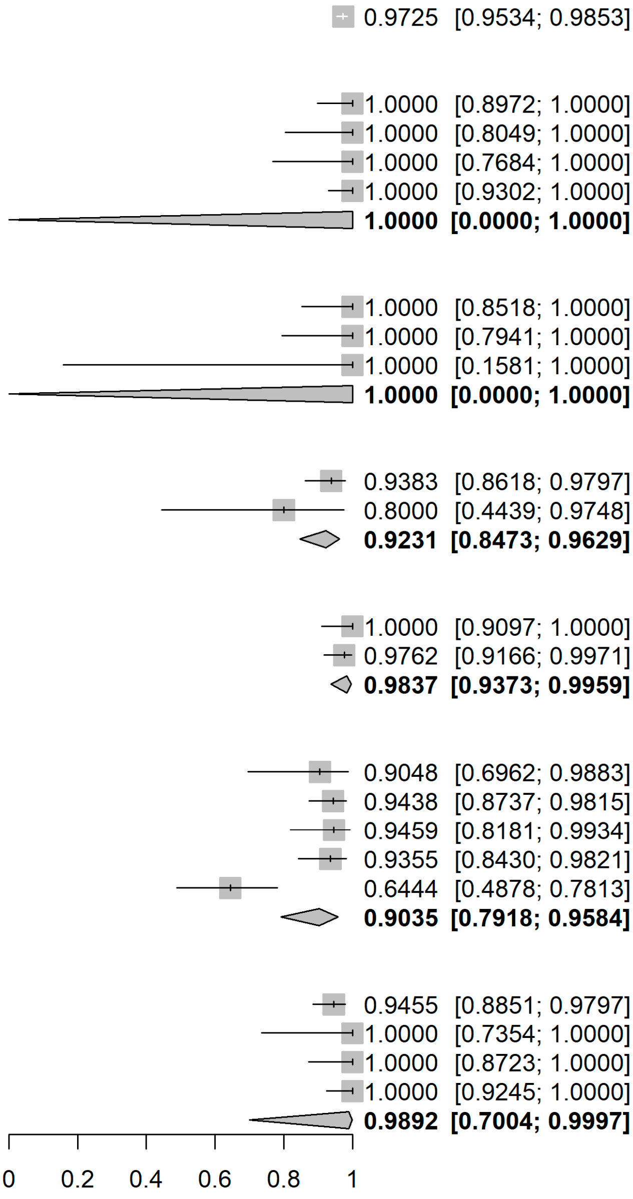

Supplementary Figure S3. PPV of the different tests in T21.

Study	TN	TN + FN	NPV	95% CI
**GeneTech**				
Sakaki 2021	43790	43791	1.0000	[0.9999; 1.0000]
**Harmony**				
Conotte 2022	865	866	0.9988	[0.9936; 1.0000]
Wergifosse 2021	3097	3097	1.0000	[0.9988; 1.0000]
Perez 2014	568	568	1.0000	[0.9935; 1.0000]
Willems 2014	2916	2917	0.9997	[0.9981; 1.0000]
**Group estimate:**		**7448**	**0.9997**	**[0.9989; 0.9999]**
**NIFTY**				
Lau 2014	1958	1958	1.0000	[0.9981; 1.0000]
Jiang 2012	887	887	1.0000	[0.9958; 1.0000]
Van 2015	681	681	1.0000	[0.9946; 1.0000]
**Group estimate:**		**3526**	**1.0000**	**[0.0000; 1.0000]**
**Panorama**				
Bajka 2022	7468	7468	1.0000	[0.9995; 1.0000]
Verma 2015	489	489	1.0000	[0.9925; 1.0000]
**Group estimate:**		**7957**	**1.0000**	**[0.0000; 1.0000]**
**PrenaTest**				
Stumm 2013	477	478	0.9979	[0.9884; 0.9999]
Hofmann 2014	5516	5516	1.0000	[0.9993; 1.0000]
**Group estimate:**		**5994**	**0.9998**	**[0.9912; 1.0000]**
**Vanadis**				
Gormus 2021	827	827	1.0000	[0.9955; 1.0000]
Palomaki 2022	2260	2261	0.9996	[0.9975; 1.0000]
Conotte 2022	863	863	1.0000	[0.9957; 1.0000]
Pooh 2021	1145	1146	0.9991	[0.9951; 1.0000]
Saidel 2023	8066	8067	0.9999	[0.9993; 1.0000]
**Group estimate:**		**13164**	**0.9998**	**[0.9993; 0.9999]**
**Verifi**				
Kershberg 2015	6497	6498	0.9998	[0.9991; 1.0000]
Marchili 2015	602	602	1.0000	[0.9939; 1.0000]
Kuitunen 2016	938	938	1.0000	[0.9961; 1.0000]
Togneri 2019	952	953	0.9990	[0.9942; 1.0000]
**Group estimate:**		**8991**	**0.9998**	**[0.9991; 0.9999]**

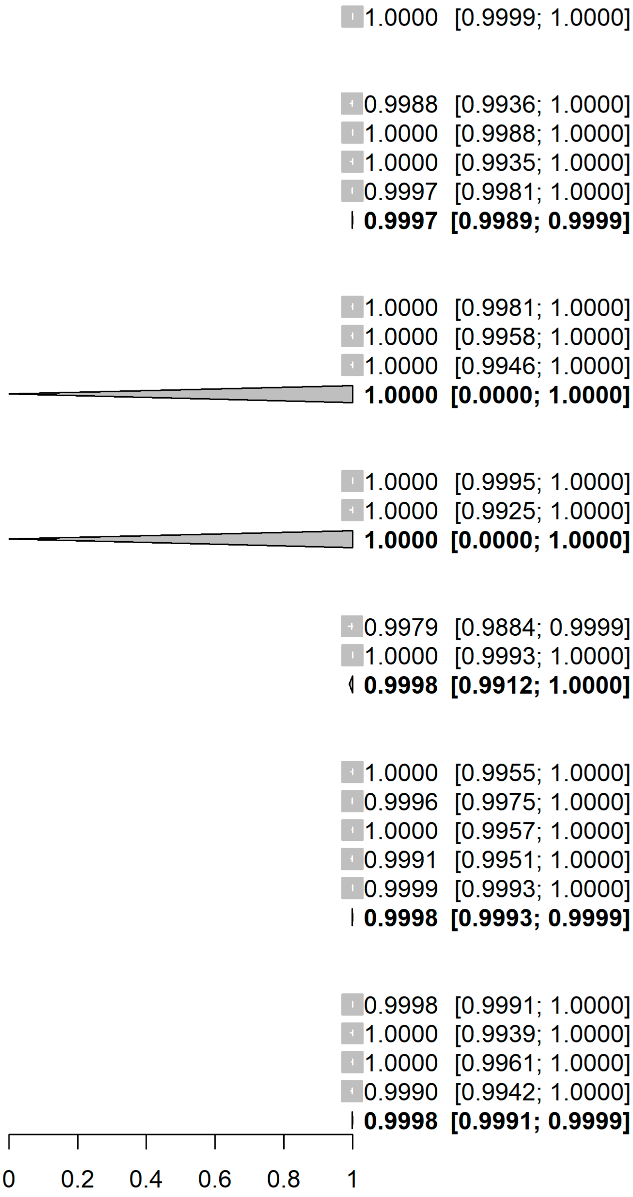

Supplementary Figure S4. NPV of the different tests in T21.

Study	TN	TN + FP	Specificity	95% CI
**GeneTech**				
Sakaki 2021	44019	44037	0.9996	[0.9994; 0.9998]
**Harmony**				
Conotte 2022	885	885	1.0000	[0.9958; 1.0000]
Wergifosse 2021	3107	3107	1.0000	[0.9988; 1.0000]
Perez 2014	579	579	1.0000	[0.9936; 1.0000]
Willems 2014	2963	2963	1.0000	[0.9988; 1.0000]
**Group estimate:**		**7534**	**1.0000**	**[0.0000; 1.0000]**
**NIFTY**				
Lau 2014	1977	1977	1.0000	[0.9981; 1.0000]
Jiang 2012	890	891	0.9989	[0.9938; 1.0000]
Van 2015	682	682	1.0000	[0.9946; 1.0000]
**Group estimate:**		**3550**	**0.9997**	**[0.9980; 1.0000]**
**Panorama**				
Bajka 2022	7529	7530	0.9999	[0.9993; 1.0000]
Verma 2015	498	498	1.0000	[0.9926; 1.0000]
**Group estimate:**		**8028**	**0.9999**	**[0.9991; 1.0000]**
**PrenaTest**				
Stumm 2013	509	509	1.0000	[0.9928; 1.0000]
Hofmann 2014	5578	5583	0.9991	[0.9979; 0.9997]
**Group estimate:**		**6092**	**0.9992**	**[0.9980; 0.9997]**
**Vanadis**				
Gormus 2021	833	837	0.9952	[0.9878; 0.9987]
Palomaki 2022	2306	2321	0.9935	[0.9894; 0.9964]
Conotte 2022	885	885	1.0000	[0.9958; 1.0000]
Pooh 2021	1156	1162	0.9948	[0.9888; 0.9981]
Saidel 2023	8092	8100	0.9990	[0.9981; 0.9996]
**Group estimate:**		**13305**	**0.9974**	**[0.9939; 0.9989]**
**Verifi**				
Kershberg 2015	6557	6568	0.9983	[0.9970; 0.9992]
Marchili 2015	611	612	0.9984	[0.9909; 1.0000]
Kuitunen 2016	951	954	0.9969	[0.9908; 0.9994]
Togneri 2019	994	995	0.9990	[0.9944; 1.0000]
**Group estimate:**		**9129**	**0.9982**	**[0.9971; 0.9989]**

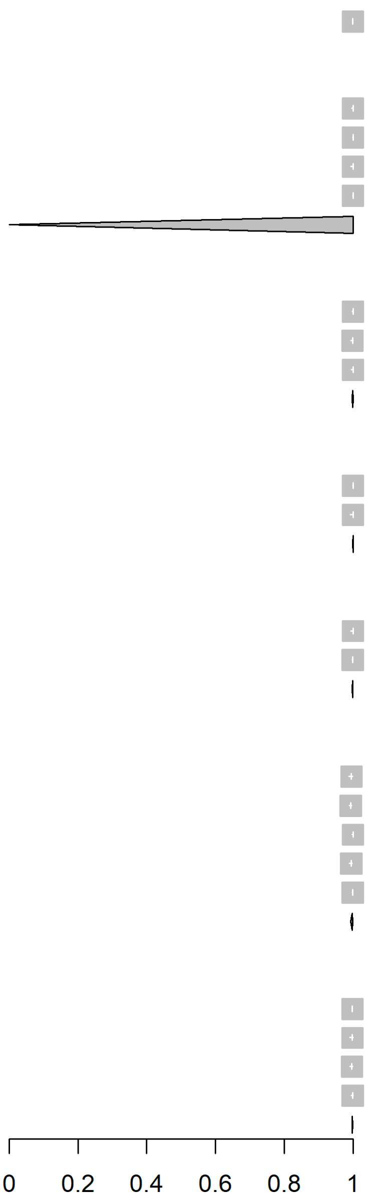

Supplementary Figure S5. Specificity of the different tests in T18.

Study	TP	TP + FN	Sensitivity	95% CI
**GeneTech**				
Sakaki 2021	224	226	0.9912	[0.9684; 0.9989]
**Harmony**				
Conotte 2022	11	15	0.7333	[0.4490; 0.9221]
Wergifosse 2021	5	7	0.7143	[0.2904; 0.9633]
Perez 2014	3	3	1.0000	[0.2924; 1.0000]
Willems 2014	4	5	0.8000	[0.2836; 0.9949]
**Group estimate:**		**30**	**0.7667**	**[0.5850; 0.8845]**
**NIFTY**				
Lau 2014	4	4	1.0000	[0.3976; 1.0000]
Jiang 2012	12	12	1.0000	[0.7354; 1.0000]
Van 2015	1	1	1.0000	[0.0250; 1.0000]
**Group estimate:**		**17**	**1.0000**	**[0.0000; 1.0000]**
**Panorama**				
Bajka 2022	19	19	1.0000	[0.8235; 1.0000]
Verma 2015	1	1	1.0000	[0.0250; 1.0000]
**Group estimate:**		**20**	**1.0000**	**[0.0000; 1.0000]**
**PrenaTest**				
Stumm 2013	8	8	1.0000	[0.6306; 1.0000]
Hofmann 2014	17	17	1.0000	[0.8049; 1.0000]
**Group estimate:**		**25**	**1.0000**	**[0.0000; 1.0000]**
**Vanadis**				
Gormus 2021	11	11	1.0000	[0.7151; 1.0000]
Palomaki 2022	28	29	0.9655	[0.8224; 0.9991]
Conotte 2022	14	15	0.9333	[0.6805; 0.9983]
Pooh 2021	46	46	1.0000	[0.9229; 1.0000]
Saidel 2023	12	12	1.0000	[0.7354; 1.0000]
**Group estimate:**		**113**	**0.9823**	**[0.9320; 0.9956]**
**Verifi**				
Kershberg 2015	38	40	0.9500	[0.8308; 0.9939]
Marchili 2015	2	2	1.0000	[0.1581; 1.0000]
Kuitunen 2016	11	11	1.0000	[0.7151; 1.0000]
Togneri 2019	5	5	1.0000	[0.4782; 1.0000]
**Group estimate:**		**58**	**0.9655**	**[0.8723; 0.9914]**

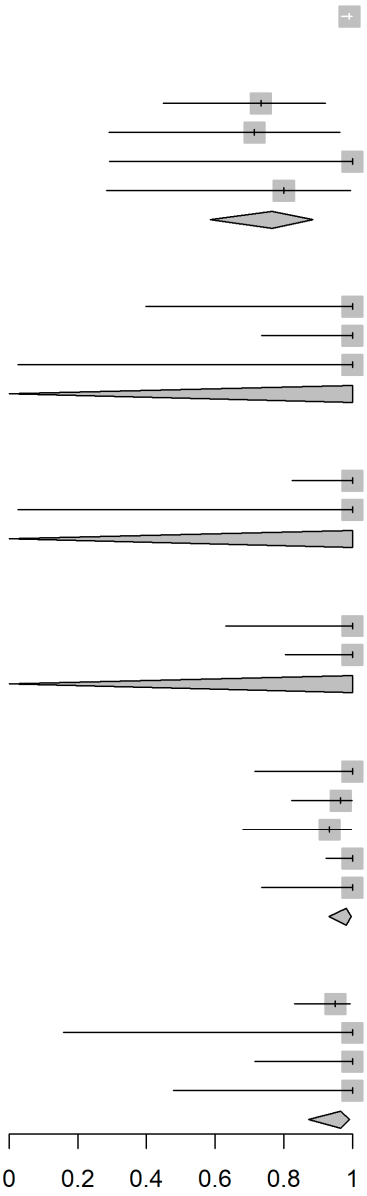

Supplementary Figure S6. Sensitivity of the different tests in T18.

Study	TP	TP + FP	PPV	95% CI
**GeneTech**				
Sakaki 2021	224	242	0.9256	[0.8850; 0.9553]
**Harmony**				
Conotte 2022	11	11	1.0000	[0.7151; 1.0000]
Wergifosse 2021	5	5	1.0000	[0.4782; 1.0000]
Perez 2014	3	3	1.0000	[0.2924; 1.0000]
Willems 2014	4	4	1.0000	[0.3976; 1.0000]
**Group estimate:**		**23**	**1.0000**	**[0.0000; 1.0000]**
**NIFTY**				
Lau 2014	4	4	1.0000	[0.3976; 1.0000]
Jiang 2012	12	13	0.9231	[0.6397; 0.9981]
Van 2015	1	1	1.0000	[0.0250; 1.0000]
**Group estimate:**		**18**	**0.9444**	**[0.6935; 0.9922]**
**Panorama**				
Bajka 2022	19	20	0.9500	[0.7513; 0.9987]
Verma 2015	1	1	1.0000	[0.0250; 1.0000]
**Group estimate:**		**21**	**0.9524**	**[0.7286; 0.9933]**
**PrenaTest**				
Stumm 2013	8	8	1.0000	[0.6306; 1.0000]
Hofmann 2014	17	22	0.7727	[0.5463; 0.9218]
**Group estimate:**		**30**	**0.8333**	**[0.6568; 0.9289]**
**Vanadis**				
Gormus 2021	11	15	0.7333	[0.4490; 0.9221]
Palomaki 2022	28	43	0.6512	[0.4907; 0.7899]
Conotte 2022	14	14	1.0000	[0.7684; 1.0000]
Pooh 2021	46	52	0.8846	[0.7656; 0.9565]
Saidel 2023	12	20	0.6000	[0.3605; 0.8088]
**Group estimate:**		**144**	**0.7937**	**[0.6236; 0.8993]**
**Verifi**				
Kershberg 2015	38	49	0.7755	[0.6338; 0.8823]
Marchili 2015	2	3	0.6667	[0.0943; 0.9916]
Kuitunen 2016	11	14	0.7857	[0.4920; 0.9534]
Togneri 2019	5	6	0.8333	[0.3588; 0.9958]
**Group estimate:**		**72**	**0.7778**	**[0.6676; 0.8592]**

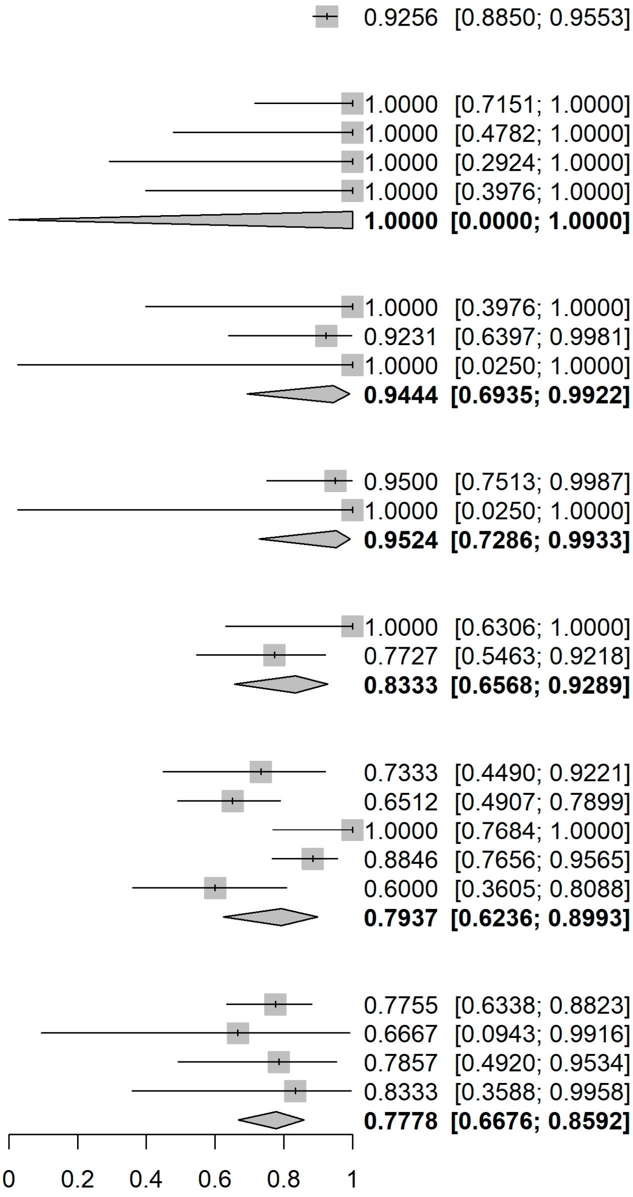

Supplementary Figure S7. PPV of the different tests in T18.

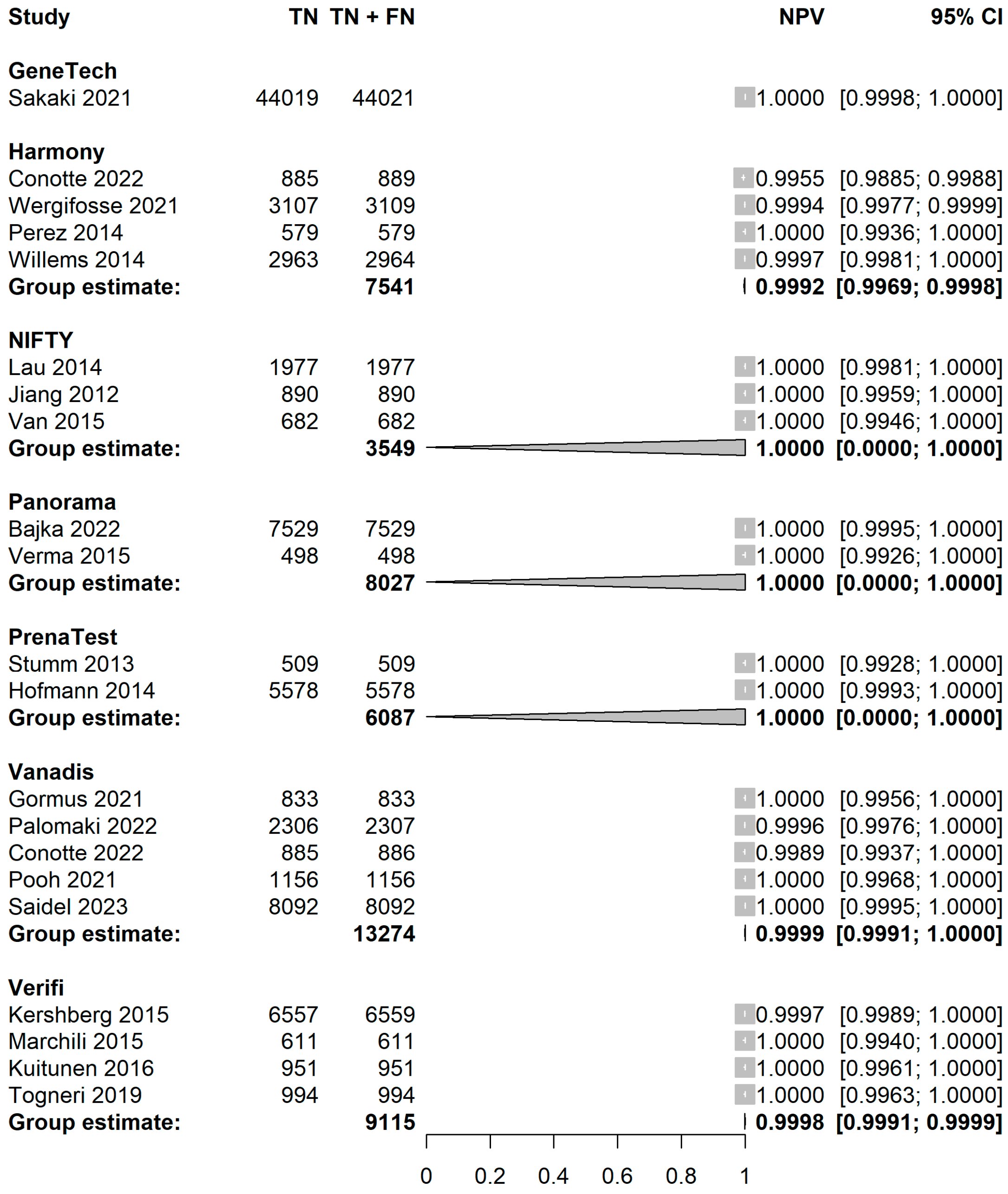

Supplementary Figure S8. NPV of the different tests in T18.

Study	TN	TN + FP	Specificity	95% CI
**GeneTech**				
Sakaki 2021	44206	44234	0.9994	[0.9991; 0.9996]
**Harmony**				
Conotte 2022	894	897	0.9967	[0.9903; 0.9993]
Wergifosse 2021	3109	3113	0.9987	[0.9967; 0.9996]
Perez 2014	582	582	1.0000	[0.9937; 1.0000]
Willems 2014	2966	2966	1.0000	[0.9988; 1.0000]
**Group estimate:**		**7558**	**0.9993**	**[0.9962; 0.9999]**
**NIFTY**				
Lau 2014	1979	1979	1.0000	[0.9981; 1.0000]
Jiang 2012	901	901	1.0000	[0.9959; 1.0000]
Van 2015	683	683	1.0000	[0.9946; 1.0000]
**Group estimate:**		**3563**	**1.0000**	**[0.0000; 1.0000]**
**Panorama**				
Bajka 2022	7531	7534	0.9996	[0.9988; 0.9999]
Verma 2015	498	498	1.0000	[0.9926; 1.0000]
**Group estimate:**		**8032**	**0.9996**	**[0.9988; 0.9999]**
**PrenaTest**				
Stumm 2013	512	512	1.0000	[0.9928; 1.0000]
Hofmann 2014	5594	5595	0.9998	[0.9990; 1.0000]
**Group estimate:**		**6107**	**0.9998**	**[0.9988; 1.0000]**
**Vanadis**				
Gormus 2021	837	839	0.9976	[0.9914; 0.9997]
Palomaki 2022	2340	2342	0.9991	[0.9969; 0.9999]
Conotte 2022	896	897	0.9989	[0.9938; 1.0000]
Pooh 2021	1196	1196	1.0000	[0.9969; 1.0000]
Saidel 2023	8102	8110	0.9990	[0.9981; 0.9996]
**Group estimate:**		**13384**	**0.9990**	**[0.9983; 0.9994]**
**Verifi**				
Kershberg 2015	6587	6593	0.9991	[0.9980; 0.9997]
Marchili 2015	614	614	1.0000	[0.9940; 1.0000]
Kuitunen 2016	962	962	1.0000	[0.9962; 1.0000]
Togneri 2019	986	988	0.9980	[0.9927; 0.9998]
**Group estimate:**		**9157**	**0.9991**	**[0.9983; 0.9996]**

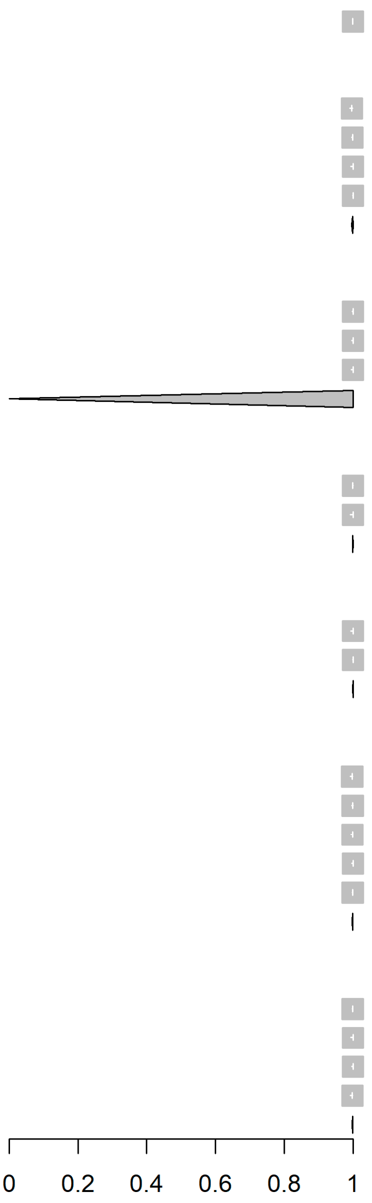

Supplementary Figure S9. Specificity of the different tests in T13.

Study	TP	TP + FN	Sensitivity	95% CI
**GeneTech**				
Sakaki 2021	29	29	1.0000	[0.8806; 1.0000]
**Harmony**				
Conotte 2022	3	3	1.0000	[0.2924; 1.0000]
Wergifosse 2021	1	1	1.0000	[0.0250; 1.0000]
Willems 2014	2	2	1.0000	[0.1581; 1.0000]
**Group estimate:**		**6**	**1.0000**	**[0.0000; 1.0000]**
**NIFTY**				
Lau 2014	2	2	1.0000	[0.1581; 1.0000]
Jiang 2012	2	2	1.0000	[0.1581; 1.0000]
**Group estimate:**		**4**	**1.0000**	**[0.0000; 1.0000]**
**Panorama**				
Bajka 2022	15	15	1.0000	[0.7820; 1.0000]
Verma 2015	1	1	1.0000	[0.0250; 1.0000]
**Group estimate:**		**16**	**1.0000**	**[0.0000; 1.0000]**
**PrenaTest**				
Stumm 2013	5	5	1.0000	[0.4782; 1.0000]
Hofmann 2014	5	5	1.0000	[0.4782; 1.0000]
**Group estimate:**		**10**	**1.0000**	**[0.0000; 1.0000]**
**Vanadis**				
Gormus 2021	9	9	1.0000	[0.6637; 1.0000]
Palomaki 2022	5	8	0.6250	[0.2449; 0.9148]
Conotte 2022	3	3	1.0000	[0.2924; 1.0000]
Pooh 2021	12	12	1.0000	[0.7354; 1.0000]
Saidel 2023	2	2	1.0000	[0.1581; 1.0000]
**Group estimate:**		**34**	**0.9881**	**[0.1760; 1.0000]**
**Verifi**				
Kershberg 2015	15	15	1.0000	[0.7820; 1.0000]
Kuitunen 2016	3	3	1.0000	[0.2924; 1.0000]
Togneri 2019	12	12	1.0000	[0.7354; 1.0000]
**Group estimate:**		**30**	**1.0000**	**[0.0000; 1.0000]**

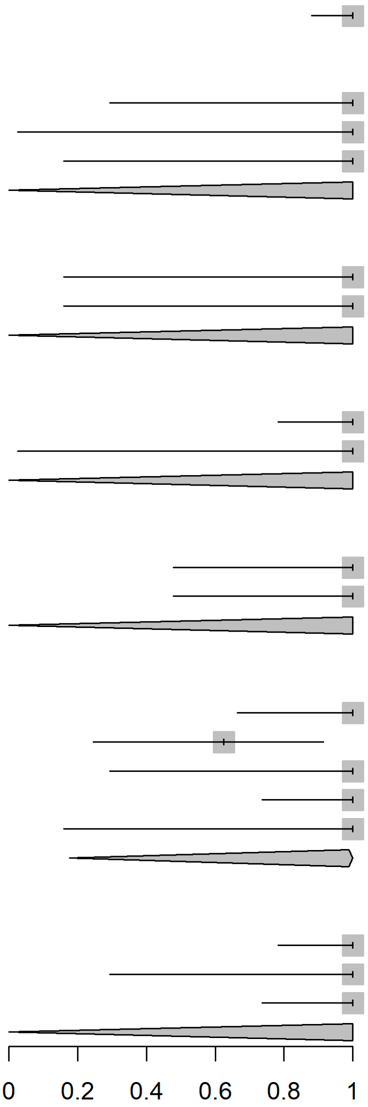

Supplementary Figure S10. Sensitivityity of the different tests in T13.

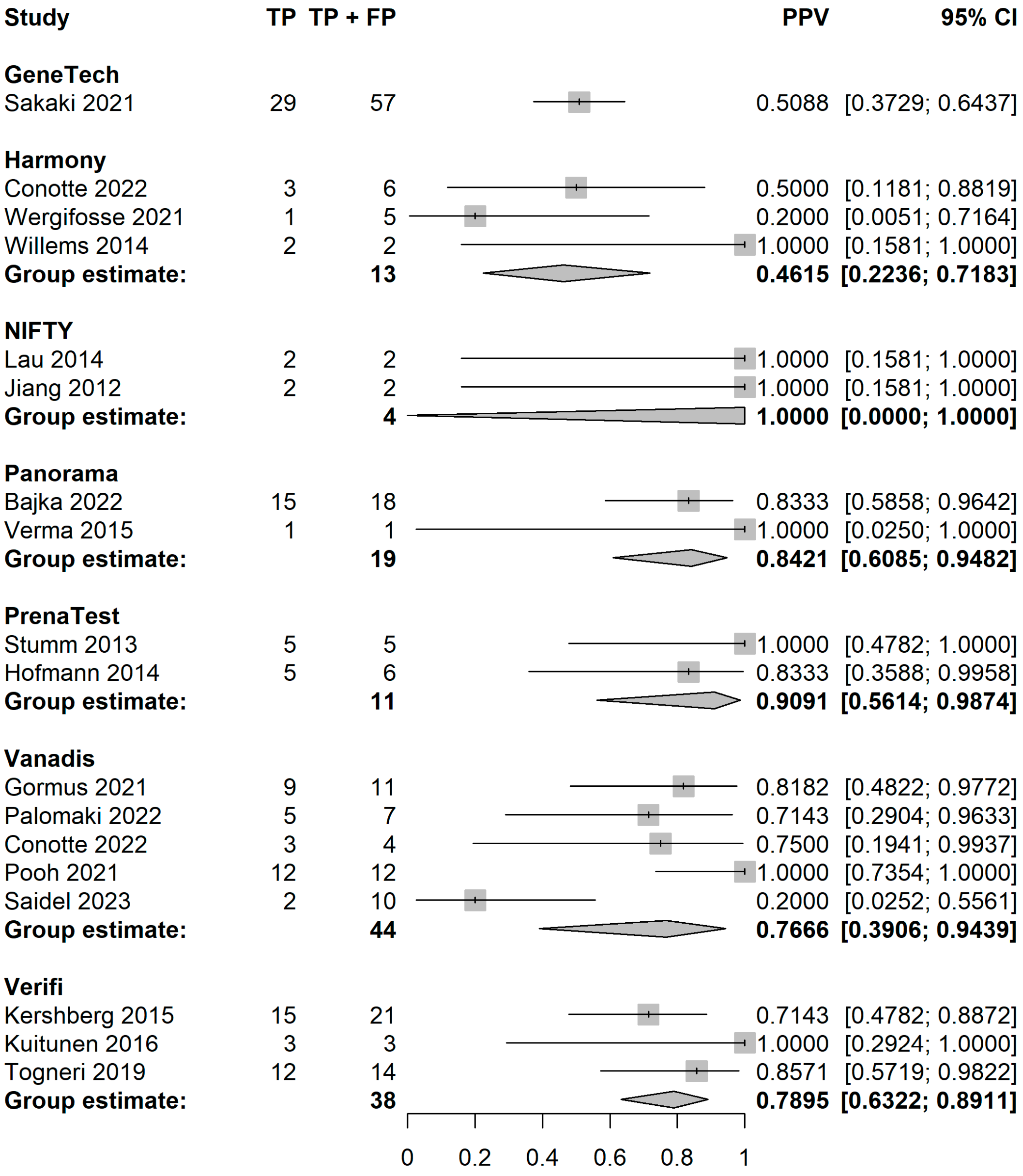

Supplementary Figure S11. PPV of the different tests in T13.

Study	TN	TN + FN	NPV	95% CI
**GeneTech**				
Sakaki 2021	44206	44206	1.0000	[0.9999; 1.0000]
**Harmony**				
Conotte 2022	894	894	1.0000	[0.9959; 1.0000]
Wergifosse 2021	3109	3109	1.0000	[0.9988; 1.0000]
Perez 2014	582	582	1.0000	[0.9937; 1.0000]
Willems 2014	2966	2966	1.0000	[0.9988; 1.0000]
**Group estimate:**		**7551**	**1.0000**	**[0.0000; 1.0000]**
**NIFTY**				
Lau 2014	1979	1979	1.0000	[0.9981; 1.0000]
Jiang 2012	901	901	1.0000	[0.9959; 1.0000]
Van 2015	683	683	1.0000	[0.9946; 1.0000]
**Group estimate:**		**3563**	**1.0000**	**[0.0000; 1.0000]**
**Panorama**				
Bajka 2022	7531	7531	1.0000	[0.9995; 1.0000]
Verma 2015	498	498	1.0000	[0.9926; 1.0000]
**Group estimate:**		**8029**	**1.0000**	**[0.0000; 1.0000]**
**PrenaTest**				
Stumm 2013	512	512	1.0000	[0.9928; 1.0000]
Hofmann 2014	5594	5594	1.0000	[0.9993; 1.0000]
**Group estimate:**		**6106**	**1.0000**	**[0.0000; 1.0000]**
**Vanadis**				
Gormus 2021	837	837	1.0000	[0.9956; 1.0000]
Palomaki 2022	2340	2343	0.9987	[0.9963; 0.9997]
Conotte 2022	896	896	1.0000	[0.9959; 1.0000]
Pooh 2021	1196	1196	1.0000	[0.9969; 1.0000]
Saidel 2023	8102	8102	1.0000	[0.9995; 1.0000]
**Group estimate:**		**13374**	**1.0000**	**[0.9904; 1.0000]**
**Verifi**				
Kershberg 2015	6587	6587	1.0000	[0.9994; 1.0000]
Marchili 2015	614	614	1.0000	[0.9940; 1.0000]
Kuitunen 2016	962	962	1.0000	[0.9962; 1.0000]
Togneri 2019	986	986	1.0000	[0.9963; 1.0000]
**Group estimate:**		**9149**	**1.0000**	**[0.0000; 1.0000]**

Supplementary Figure S12. NPV of the different tests in T13.

Test	Sensitivity	95% CI		Specificity	95% CI		PPV	
NIFTY	1,0000	0,0000	1,0000	1,0000	0,0000	1,0000	1,0000	0,00
GeneTech	0,9978	0,9487	0,9997	0,9997	0,9995	0,9998	0,9725	0,95
Verifi	0,9896	0,9593	0,9974	0,9993	0,9985	0,9997	0,9892	0,70
PrenaTest	0,9918	0,9442	0,9988	0,9997	0,9987	0,9999	0,9837	0,93
Panorama	1,0000	0,0000	1,0000	0,9991	0,9982	0,9996	0,9231	0,84
Harmony	0,9831	0,9348	0,9958	1,0000	0,0000	1,0000	1,0000	0,00
Vanadis	0,9868	0,9600	0,9958	0,9978	0,9968	0,9985	0,9035	0,79

Supplementary Table S1. Metaanalysis data for T21

Test	Sensitivity	95% CI		Specificity	95% CI		PPV	
NIFTY	1,0000	0,0000	1,0000	0,9997	0,9980	1,0000	0,9444	0,69
GeneTech	0,9912	0,9653	0,9978	0,9996	0,9994	0,9997	0,9256	0,88
Verifi	0,9655	0,8723	0,9914	0,9982	0,9971	0,9989	0,7778	0,66
PrenaTest	1,0000	0,0000	1,0000	0,9992	0,9980	0,9997	0,8333	0,65
Panorama	1,0000	0,0000	1,0000	0,9999	0,9991	1,0000	0,9524	0,72
Harmony	0,7667	0,5850	0,8845	1,0000	0,0000	1,0000	1,0000	0,00
Vanadis	0,9823	0,9320	0,9956	0,9974	0,9939	0,9989	0,7937	0,62

Supplementary Table S2. Metaanalysis data for T18

Test	Sensitivity	95% CI		Specificity	95% CI		PPV	
NIFTY	1,0000	0,0000	1,0000	1,0000	0,0000	1,0000	1,0000	0,00
GeneTech	1,0000	0,0000	1,0000	0,9994	0,9991	0,9996	0,5088	0,38
Verifi	1,0000	0,0000	1,0000	0,9991	0,9983	0,9996	0,7895	0,63
PrenaTest	1,0000	0,0000	1,0000	0,9998	0,9988	1,0000	0,9091	0,56
Panorama	1,0000	0,0000	1,0000	0,9996	0,9988	0,9999	0,8421	0,60
Harmony	1,0000	0,0000	1,0000	0,9993	0,9962	0,9999	0,4615	0,22
Vanadis	0,9881	0,1760	1,0000	0,9990	0,9983	0,9994	0,7666	0,39

Supplementary Table S3. Metaanalysis data for T13
